# Controlling Alzheimer’s Disease Through the Deep Brain Stimulation to Thalamic Relay Cells

**DOI:** 10.3389/fncom.2021.636770

**Published:** 2021-11-08

**Authors:** XiaoLi Yang, RuiXi Zhang, ZhongKui Sun, Jürgen Kurths

**Affiliations:** ^1^School of Mathematics and Statistics, Shaanxi Normal University, Xi’an, China; ^2^Department of Applied Mathematics, Northwestern Polytechnical University, Xi’an, China; ^3^Potsdam Institute for Climate Impact Research, Potsdam, Germany; ^4^Department of Physics, Humboldt University of Berlin, Berlin, Germany; ^5^Centre for Analysis of Complex Systems, World-Class Research Center “Digital Biodesign and Personalized Healthcare”, Sechenov First Moscow State Medical University, Moscow, Russia

**Keywords:** neurocomputation, power spectrum analysis, thalamo-cortico-thalamic model, deep brain stimulation, Alzheimer’s disease

## Abstract

Experimental and clinical studies have shown that the technique of deep brain stimulation (DBS) plays a potential role in the regulation of Alzheimer’s disease (AD), yet it still desires for ongoing studies including clinical trials, theoretical approach and action mechanism. In this work, we develop a modified thalamo-cortico-thalamic (TCT) model associated with AD to explore the therapeutic effects of DBS on AD from the perspective of neurocomputation. First, the neuropathological state of AD resulting from synapse loss is mimicked by decreasing the synaptic connectivity strength from the Inter-Neurons (IN) neuron population to the Thalamic Relay Cells (TRC) neuron population. Under such AD condition, a specific deep brain stimulation voltage is then implanted into the neural nucleus of TRC in this TCT model. The symptom of AD is found significantly relieved by means of power spectrum analysis and nonlinear dynamical analysis. Furthermore, the therapeutic effects of DBS on AD are systematically examined in different parameter space of DBS. The results demonstrate that the controlling effect of DBS on AD can be efficient by appropriately tuning the key parameters of DBS including amplitude A, period P and duration D. This work highlights the critical role of thalamus stimulation for brain disease, and provides a theoretical basis for future experimental and clinical studies in treating AD.

## Introduction

Alzheimer’s disease (AD), a common degenerative disease of the central neural system, is clinically characterized by language disorders, cognitive dysfunction and behavior changes. The onset of this dynamic disease is relatively slow and also difficult to detect. As the disease progresses, neurons involved in thinking, learning, memory and movement are damaged, resulting in a memory impairment and even a loss of daily behavior. There have been about 44 million people worldwide suffering from AD or related dementia in 2015. It is estimated that 4.6 million people will have dementia each year and the number will double by 2030 (Prince et al., 2013). The rapid increase of AD patients has brought a heavy burden on families and society, which motivates more and more scientists and physicians to explore more the pathogenesis, diagnosis and treatment of AD.

At present, the treatments for patients with AD are quite limited. The main pharmacologic treatments include memantine, N-methyl-D-aspartate receptor antagonist and acetylcholinesterase inhibitors (Thies and Bleiler, 2011). The therapeutic effect of these treatments is not satisfactory and the cost of drug research investment is high. Thus, it is urgent to develop alternative treatments for AD (Hamani et al., 2008; Laxton and Lozano, 2013). Deep brain stimulation (DBS), as one kind of invasive brain stimulation, has attracted extensive academic and clinical attention for treating many central nervous system diseases (Moro and Lang, 2006; Velasco et al., 2007; Zhong et al., 2011; Suarez-Cedeno et al., 2017; Fan et al., 2020; Yu et al., 2020). The DBS system consists of one or more electrodes and a pulse generator extension cord (Miocinovic et al., 2013). The electrodes are stereoscopically implanted into a specific nerve nucleus in the brain, and then a pulse signal of a certain frequency is released to stimulate and modulate the neurons near the electrode, thereby alleviating the symptoms of the disease. In the last 30 years, DBS implants have been performed in more than 120,000 patients all over the world with demonstrated benefit in Parkinson’s disease, obsessive compulsive disorder, tremor, depression and dystonia. As for the DBS treatment of Parkinson’s disease, the two primary targets are globus pallidus pars interna (GPi) and sub-thalamic nucleus (STN). GPi-DBS can reduce motor complications such as dyskinesia, while STN-DBS usually helps to reduce the need for dopamine replacement medications and improve the cardinal motor features of Parkinson’s disease (Jakobs et al., 2020).

Preliminary research has shown promising effects of DBS in the field of AD. Targeting the brain region of the nucleus basalis of Meynert (NBM), Turnbull et al. reported a DBS trial on a 74-year-old moderate AD male patient in 1985, where although they did not find clinical improvement of memory or cognition, hopefully by positron emission tomography (PET) scans they detected that the cortical glucose metabolic activity in the left parietal and left temporal lobes was preserved and the deterioration in the left frontal area was partially stopped (Turnbull et al., 1985). This was the first case of DBS treatment on AD patients in the world, which inspired further exploration of DBS trial on AD in human clinical and animal experiments. For example, using the fornix as the targeted brain region, Laxton et al. conducted a clinical trial of DBS on six patients with mild Alzheimer’s disease who were receiving drug treatment (Laxton et al., 2010). PET scans showed that the impaired glucose utilization in the temporal lobe and parietal lobe was significantly reversed after 12 months of stimulation. Evaluation of the Alzheimer’s Disease Assessment Scale, Cognitive Subscale (ADAS-Cog) and the Mini Mental State Examination (MMSE) suggested possible improvements and/or slowing of cognitive decline at 6 and 12 months in some patients. The results indicated that DBS does not only activate the brain’s default mode network, but also it can drive neural activity in the memory circuit including the entorhinal and hippocampal areas. Smith et al. performed DBS of the fornix by selecting 5 mild AD patients with an average age of 62.6 years old (Smith et al., 2012). PET studies showed that DBS would increase cerebral glucose metabolism in cortical and hippocampal circuits after 1 year. Fontaine et al. selected 1 mild AD patient who fully met the inclusion criteria among 110 AD patients, after 1 year’s DBS treatment of bilateral fornix the memory scores including MMSE, ADAS-Cog, Free and Cued Selective Reminding Test were stabilized compared to baseline, while PET examination showed the mesial temporal lobes metabolism was increased (Fontaine et al., 2013). In order to check safety and efficacy of DBS in the treatment of dementia, Lozano et al. performed a phase II study of fornix DBS in a randomized, double-blind trial in 42 subjects with mild AD (Lozano et al., 2016). The cognitive function and cerebral glucose metabolism were, respectively measured by ADAS-Cog and PET imaging up to 12 months post-implantation. Three important results were reported. The first one is that surgical intervene and DBS were safe and well tolerated. The second one is that there were no obvious differences in cognitive outcomes in DBS ON versus OFF participants at 12 months as a whole. Interestingly, patients receiving DBS showed increased glucose metabolism at 6 months but this was not significant at 12 months. The last one is the remarkable interaction between treatment outcome and age by *post-hoc* analysis, i.e., subjects aged ≥65 years may have derived benefit on a greater increase in cerebral glucose metabolism and clinical outcomes, while patients <65 years may have trended toward being worse with DBS treatment. Mann et al. investigated a DBS treatment of entorhinal cortex in 3xTg mice and found that the memory of these mice was improved, which further supported that DBS may be as a potential therapeutic technique for AD (Mann et al., 2018). In addition, different targets of the brain in animal models such as fornix (Hescham et al., 2013b), NBM (Hotta et al., 2009) and entorhinal cortex (Stone et al., 2011) were implicated for DBS trails. Neuroanatomical, neurochemical and neurophysiological changes within the memory circuits were discussed in preclinical studies, which indicated enhancement of memory function (Aldehri et al., 2018). Some works also attempted to elucidate the underlying mechanisms of DBS action, for example, the enhanced synaptic plasticity and long-term potentiation (Hao et al., 2015), neurogenesis (Chamaa et al., 2016), increased neurotransmitter release (Hescham et al., 2016), hippocampal enlargement (Sankar et al., 2015) and release of growth factors and neurotransmitter respecification (Hescham et al., 2013a) have been described in preclinical studies. Different study designs, population, patients selection or samples sizes make drawing consistent conclusions rather difficult. Interested readers are referred to the recent reviews and references wherein (Aldehri et al., 2018; Chang et al., 2018). The relevant action mechanisms of DBS are still far from being understood. The effect of DBS on AD is somewhat optimistic, DBS treatments for AD are however far from mature at present. In the future, not only standardized experimental design with larger sample size and longitudinal follow-up, but also an analytical approach of the DBS control is desired for clinical guidance and application (Chang et al., 2018).

Note that thalamus lesion is significantly correlated with AD, and the connection between the thalamus and the near brain region such as hippocampus is closely related to cognitive impairment and memory loss (Johansen-Berg et al., 2005; Aggleton et al., 2010; Zarei et al., 2010; Zhang et al., 2010). The neuroimaging technology of structural MRI and diffusion tensor has detected abnormal white matter fibers and structural atrophy in the thalamus of AD patients (Zarei et al., 2010). Thus, stimulation of the thalamus has gradually aroused researchers’ attention in the DBS treatments of AD. Recently, Zhang et al. performed DBS over the anterior nucleus of thalamus, entorhinal cortex and fornix of rats with AD, in which they found that the hippocampal-dependent spatial memory was greatly promoted by these stimulations (Zhang et al., 2015). Through implementing DBS treatment of the midline thalamic nucleus on AD mice, the result exhibited that the short-term memory of the hippocampal CA1 area can be enhanced by tuning the stimulation frequency and intensity (Arrieta-Cruz et al., 2010). Meanwhile, targeting the nerve nuclei of the thalamus region has also attracted interest in the DBS treatments of epilepsy (Hu and Wang, 2015; Hu et al., 2017; Wang and Wang, 2017). Motivated by the above scientific findings, this work localizes the thalamic relay nucleus, an important nerve nuclei in the thalamus, as the target region in the research of DBS treatment.

On the other hand, it is well known that pathological changes of some brain region can induce abnormalities in oscillations of field potentials recorded by electroencephalograms (EEG). EEG markers have been considered as a valuable tool in disease differential/diagnosis, drugs safety and therapy efficacy (Jeong, 2004; Dauwels et al., 2010). As for AD, previous works in the literature have indicated that the early stage of AD is characterized by a decrease power of EEG in the beta and alpha band, but an increase power of EEG in theta band. This leads to a reducing of the total power of the EEG spectrum (Coben et al., 1985; Brenner et al., 1986; Giaquinto and Nolfe, 1986; Prinz and Vitiell, 1989; Schreiter-Gasser et al., 1993; Jeong, 2004). Meanwhile, the hallmark of EEG reversals in AD patients who received a treatment were also explored by conventional spectral analysis. For example, Knott et al. implemented acute nicotine administration on AD patients and examined the drug effect by spectrum-analyzed EEG (Knott et al., 2000). The results demonstrated that nicotine can significantly shift EEG toward normal values by reducing slow wave power within the delta and theta band and augmenting fast wave power within the alpha and beta band. Also, Balkan et al. examined the effects of donepezil on probable AD patients by EEG spectral analysis (Balkan et al., 2003). The results indicated that donepezil can exert a positive effect on EEG in AD by decreasing delta activity and increasing alpha and beta activity. By a neuropsychological and computerized EEG study, Agnoli et al. examined the effect of cholinergic and anticholinergic drugs on AD patients (Agnoli et al., 1983). They found that all acute administration of some cholinergic drugs, such as physostigmine edrophonium chloride, can improve attention and memory performances, where the tendency of EEG spectral analysis shifted into more normal patterns matching with the subjects’ age. The reversal of EEG in these comparative tests of drug effects for AD is obvious, i.e., the reducing of the slow wave power within the delta/theta band as well as the augmenting of the fast wave power within the alpha/beta band is a notable tendency of EEG shift to normal values. To our most knowledge, until now EEG rhythm has not been applied to the clinical evaluation of DBS for AD. This work attempts to employ the power spectral analysis of EEG rhythm to evaluate the controlling effect of DBS on AD from the perspective of neurocomputation, which may provide a theoretical basis for future clinical DBS studies in treating AD.

The previous clinical practices and animal experiments of DBS trials have implied that deep brain stimulation may be a potential therapeutic strategy to modulate AD brain activity (Zhang et al., 2015; Aldehri et al., 2018; Chang et al., 2018; Mann et al., 2018). Regretfully, how to localize the specific target in the brain region together with choosing the appropriate stimulation parameters is still an intractable problem in medicine. In addition, the relevant action mechanisms of DBS are far from being understood. Thus, this work develops a neural mass model defining the thalamo-cortico-thalamic (TCT) circuitry at a realistic population scale, whereby theoretically exploring the therapeutic effects of DBS on AD in a virtual simulating environment. First, the modified TCT model is employed to simulate the neuropathological state of AD by decreasing one synaptic connectivity from the IN neuron population to the TRC neuron population. Then we attempt to implant a certain deep brain stimulation voltage into the neural nuclei of TRC in this TCT model. Based on the techniques of power spectrum analysis and nonlinear dynamical analysis, the therapeutic effects of DBS on AD are theoretically examined through adjusting the key parameters of DBS. In the following, the modified TCT model together with the simulation method is presented in section “Model Presentation and Preliminary.” Section “Main Results” presents the main results about the controlling effect of DBS on AD. A brief conclusion and discussion are given in section “Conclusion and Discussion.”

## Model Presentation and Preliminary

### A Modified Thalamo-Cortico-Thalamic Model With Deep Brain Stimulation Voltage

**FIGURE 1:**
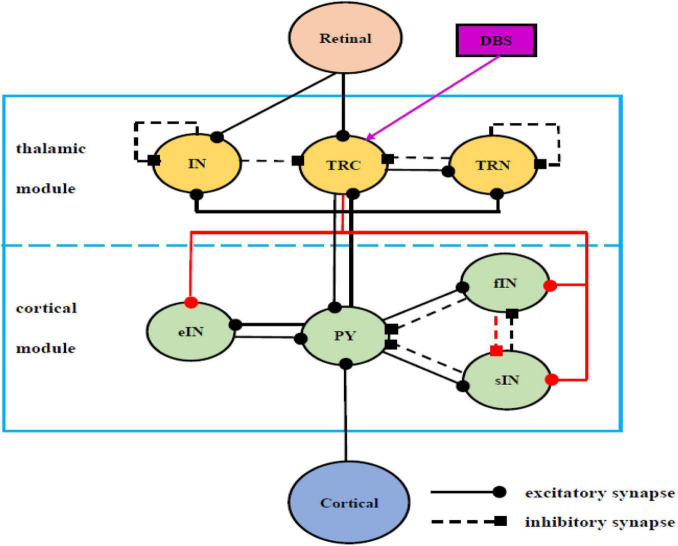
Schematic framework and synaptic structures of the modified TCT model with DBS voltage on the basis of previous works (Bhattacharya et al., 2011c, 2014; Li et al., 2020), which in turn are sourced from Wendling et al. (2002); Sherman (2006), Zavaglia et al. (2006); Pi et al. (2013), Cona et al. (2014), and Jiang et al. (2015). The solid line with the round head represents excitatory synapses, and the dashed line with square head represents inhibitory synapses. The purple of DBS voltage, a newly introduced device, has been implanted into the TRC cell population compared with the work (Li et al., 2020).

It is well accepted that a large part of the alpha rhythm may originate *via* cortico-thalamic interaction (Steriade and Deschenes, 1984; Steriade et al., 1991; Hughes and Crunelli, 2005). In the literature some sophisticate models of the interaction between the thalamus and the cortex have been developed. The Alpha Rhythm model (ARm), proposed by da Silva et al. (1974), consists of two populations of the interneurons (IN) and the thalamocortical relay cells (TRC) of the Lateral Geniculate Nucleus (LGN) interconnected through negative feedback. The ARm is a classical neural-mass model to define the EEG alpha rhythmic activity of the thalamo-cortical circuitry (da Silva et al., 1974). Subsequently, this model was appropriately modified and extensively investigated by incorporating additional mechanisms and further synapses, to mimic not only alpha, but also beta, theta and gamma band rhythms (Zavaglia et al., 2006; Pons et al., 2010; Ursino et al., 2010; Cona et al., 2014). Based on the seminal model of ARm, the correlations between brain rhythm and synapse connectivity were studied in the context of AD (Bhattacharya et al., 2010a,b, 2011a) which indicated that the increase of inhibitory connectivity plays an important role on slowing of alpha rhythmic oscillation. Recently, a modified ARm (Bhattacharya et al., 2011b, 2013) was developed by replacing the inhibitory IN population in the ARm with Thalamic Reticular Necleus (TRN), whose synaptic connectivity was also updated according to recent experimental data obtained from LGN of the cat and the rat (Horn et al., 2000; Jones, 2007). Then, based on the biological experimental data sourced from the dorsal LGN of the rodent and mammalian thalamus, a thalamo-cortico-thalamic loop with more biologically plausible synaptic organization was further modeled (i.e., the TCT model), in which how the various connectivity pathways influenced the dominant frequency and the alpha band power were explored (Bhattacharya et al., 2011c, 2014). The previous biological experimental results indicated that there are mutual effects between different inhibitory neurons, thus forming a basic disinhibitory circuit in the mammalian cerebral (Pi et al., 2013; Jiang et al., 2015). Meanwhile, the TRC of the thalamus transmits excitatory sensory information from the periphery to the cortex areas (Cona et al., 2014). On the basis of these two important biologically plausible ingredients and the TCT model (Bhattacharya et al., 2011c, 2014), our recent work modified the TCT model (i.e., the modified TCT model) by incorporating four new synapse connection (Li et al., 2020). One new inhibitory pathway from the fIN neuron population to the sIN neuron population is introduced to construct the disinhibitory circuit in the cortical module representing the disinhibition property between different inhibitory interneurons. The rest three new pathways are from the TRC neurons to the eIN, fIN and sIN neuron population to achieve the full relay function of the thalamic relay nucleus to the cortical region. The results revealed that the decreased connectives of some new synapse pathways and some original synapse pathways were associated with abnormal brain alpha rhythm oscillations of AD (Li et al., 2020). The present work focuses on controlling AD through the deep brain stimulation, thus this modified TCT model is further extended by newly introducing a device of DBS voltage to the neural nuclei of TRC. As illustrated in the previous works (Bhattacharya et al., 2011c, 2014; Li et al., 2020), the schematic framework and synaptic structure for this modified TCT model with DBS voltage can be outlined in Figure 1.

In detail, the model used in this work is composed of a thalamus module, a cortical module and one kind of deep brain stimulation voltage. There are Pyramid cells (PY), excitatory Inter-Neurons (eIN), slow inhibitory Inter-Neurons (sIN) and fast inhibitory Inter-Neurons (fIN) populations in the cortical module. There are TRC, IN and TRN populations in the thalamus module. TRC receives inhibitory synapses from IN and TRN and sends excitatory feedback to TRN. Meanwhile, IN and TRN produce inhibitory synapses to themselves. PY produces excitatory synapses on the three cell groups of the thalamus module. The external input of the thalamus module is the excitatory input to IN and TRC sent by the retina. Conversely, TRC produces excitatory projections on the four cell groups of the cortical module to realize its full relay function. PY generates excitatory input to eIN, sIN and fIN. In turn, eIN sends excitatory feedback to PY, and sIN and fIN send inhibitory feedback to PY. At the same time, sIN and fIN have mutually inhibitory synapses. The external input of the cortical module is the excitatory input to PY from the adjacent cortical area. Note that, a newly introduced device of the deep brain stimulation voltage is applied to TRC in this work, which is indicated by the purple arrow in Figure 1.

The modified TCT model with DBS voltage can be described by the following differential equations, which govern the dynamical behavior for all involved neuron populations:

Retinal


$${\dot{x}}_{ret1}=x_{ret2}$$

(1) $${\dot{x}}_{ret2}=\frac{H_{et}}{\tau_{et}}P_{1}\left(t\right)-\frac{2}{\tau_{et}}x_{ret2}-\frac{1}{\tau_{et}^{2}}x_{ret2}$$

IN


$$V_{in}=C_{sre}x_{ret1}+C_{spe}x_{py1}-C_{ssi}x_{in1}$$

$$S\left(V_{in}\right)=\frac{2e_{0}}{1+e^{v(s_{0-V_{in})}}}$$

$${\dot{x}}_{in1}=x_{in2}$$

(2) $${\dot{x}}_{in2}=\frac{H_{i}}{\tau_{i}}S\left(V_{in}\right)-\frac{2}{\tau_{i}}x_{in2}-\frac{1}{\tau_{i}^{2}}x_{in1}$$

TRC


$$V_{trc}=C_{tre}x_{ret1}+C_{tpe}x_{py1}-C_{tsi}x_{in1}-C_{tni}x_{trn1}+I_{DBS}R$$

$$S\left(V_{trc}\right)=\frac{2e_{0}}{1+e^{v(s_{0-V_{trc})}}}$$

$${\dot{x}}_{trc1}=x_{trc2}$$

(3) $${\dot{x}}_{trc2}=\frac{H_{et}}{\tau_{et}}S\left(V_{trc}\right)-\frac{2}{\tau_{et}}x_{trc2}-\frac{1}{\tau_{et}^{2}}x_{trc1}$$

TRN


$$V_{trn}=C_{nte}x_{trc1}+C_{npe}x_{py1}-C_{nni}x_{trn1}$$

$$S\left(V_{trn}\right)=\frac{2e_{0}}{1+e^{v(s_{0-V_{trn})}}}$$

$${\dot{x}}_{trn1}=x_{trn2}$$

(4) $${\dot{x}}_{trn2}=\frac{H_{i}}{\tau_{i}}S\left(V_{trn}\right)-\frac{2}{\tau_{i}}x_{trn2}-\frac{1}{\tau_{i}^{2}}x_{trn1}$$

eIN


$$V_{ein}=C_{bpe}x_{py1}+C_{bte}x_{trc1}$$

$$S\left(V_{ein}\right)=\frac{2e_{0}}{1+e^{v(s_{0-V_{ein})}}}$$

$${\dot{x}}_{eIN1}=x_{eIN2}$$

(5) $${\dot{x}}_{eIN2}=\frac{H_{ec}}{\tau_{ec}}S\left(V_{ein}\right)-\frac{2}{\tau_{ec}}x_{eIN2}-\frac{1}{\tau_{ec}^{2}}x_{eIN1}$$

PY


$$V_{py}=C_{pce}x_{cc1}+C_{pte}x_{trc1}+C_{pbe}x_{ein1}-C_{pli}x_{sin1}-C_{pfi}x_{fin1}$$

$$S\left(V_{py}\right)=\frac{2e_{0}}{1+e^{v(s_{0-V_{py})}}}$$

$${\dot{x}}_{py1}=x_{py2}$$

(6) $${\dot{x}}_{py2}=\frac{H_{ec}}{\tau_{ec}}S\left(V_{py}\right)-\frac{2}{\tau_{ec}}x_{py2}-\frac{1}{\tau_{ec}^{2}}x_{py1}$$

fIN


$$V_{fin}=C_{fpe}x_{py1}C_{fte}x_{trc1}-C_{fli}x_{sIN1}$$

$$S\left(V_{fin}\right)=\frac{2e_{0}}{1+e^{v(s_{0-V_{fin})}}}$$

$${\dot{x}}_{fIN1}=x_{fIN2}$$

(7) $${\dot{x}}_{fIN2}=\frac{H_{if}}{\tau_{if}}S\left(V_{fin}\right)-\frac{2}{\tau_{if}}x_{fIN2}-\frac{1}{\tau_{if}^{2}}x_{fIN1}$$

sIN


$$V_{sin}=C_{lpe}x_{py1}+C_{lte}x_{trc1}-C_{lfi}x_{fin1}$$

$$S\left(V_{sin}\right)=\frac{2e_{0}}{1+e^{v(s_{0-V_{sin})}}}$$

$${\dot{x}}_{sIN1}=x_{sIN2}$$

(8) $${\dot{x}}_{sIN2}=\frac{H_{il}}{\tau_{il}}S\left(V_{sin}\right)-\frac{2}{\tau_{il}}x_{sIN2}-\frac{1}{\tau_{il}^{2}}x_{sIN1}$$

Cortico-cortical


$${\dot{x}}_{cc1}=x_{cc2}$$

(9) $${\dot{x}}_{cc2}=\frac{H_{ec}}{\tau_{ec}}P_{2}\left(t\right)-\frac{2}{\tau_{ec}}x_{cc2}-\frac{1}{\tau_{ec}^{2}}x_{cc1}$$

**FIGURE 2:**
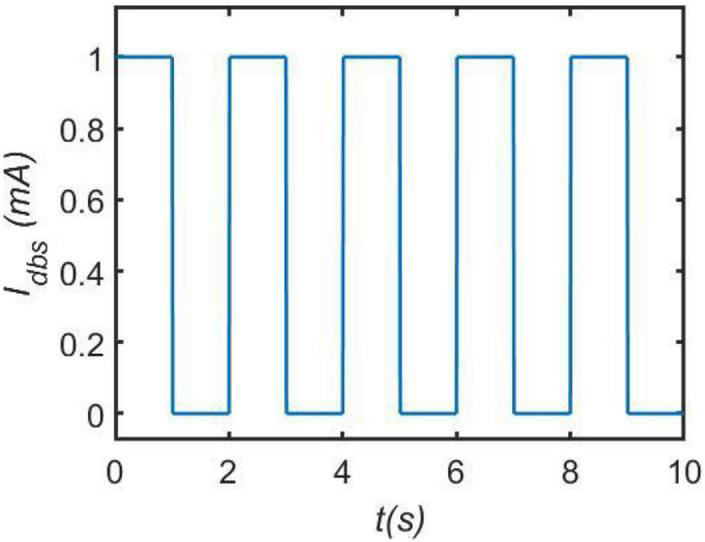
An illustrative example of current pulses DBS. Here, we set the amplitude A = 1*m*A, the period P = 2s and the duration D = 1s.

In Equation (3),I_DBS_R represents one kind of deep brain stimulation voltage, in which I_DBS_ is the DBS current and R is the resistance. In this work, the DBS current is modeled by the following square wave stimulation current (Hu and Wang, 2015; Hu et al., 2017):


(10) $${\text{I}}_{\text{DBS}}={\text{A}}^{*}H\left(\sin\left(\frac{2\pi \text{t}}{\text{P}}\right)\right)\left(1-\text{H}\left(\sin\left(\frac{2\pi \left(\text{t}+\text{D}\right)}{\text{P}}\right)\right)\right)$$

where A is the amplitude and P is the period of the DBS current, and D is the duration of positive input in one period determining duty cycle of positive pulse. H is the Heaviside step function, i.e., H(y) = 1 when y 0, and H(y) = 0 when y ⩽ 0. As indicated by the previous work (Hu and Wang, 2015; Hu et al., 2017), here R is usually set to 1Ω for simplicity. In order to understand the DBS current vividly, a specific DBS square wave is depicted in Figure 2 in the case of A = 1mA,P = 2s and D = 1s.

The external input to the thalamic module is denoted by *P*_1_(*t*), which comes from the retinal spiking neurons in an awake situation when eyes are closed. In Equation (1), *P*_1_(*t*) is simulated by Gaussian white noise with mean μ_*r*_ and variance φ_*r*_. In Equation (9), *P*_2_(*t*) represents the extrinsic input of the cortical module from the neighboring cortical regions, which is simulated by Gaussian white noise with mean μ_*c*_ and variance φ_*c*_. *H*_*et*_ and *H*_*ec*_ are the strength of the excitatory postsynaptic potential (PSP) of the thalamic and cortical modules, respectively. The strength of inhibitory PSP of the thalamus module is expressed by *H_i_*. The parameters *H*_*il*_ and *H*_*if*_ represent the strength of inhibitory PSP generated by the sIN (l) or fIN (f) in the cortical module, respectively. The time constants of the excitatory PSP of the thalamic module and cortical module is expressed by τ_et_ and τ_ec_, respectively. The time constant of the inhibitory PSP in the thalamic module is denoted by τ_*i*_. The parameters τ_*il*_ and τ_*if*_ represent the time constant of the inhibitory PSP generated by the sIN (*l*) or fIN (*f*) in the cortical module, respectively. To better despict synapse connection between different cell populations, we use different lowercase letters to denote nerve nucleis in the model. In detail,t represents TRC, n represents TRN, s represents IN, r represents retina, p represents PY,l represents sIN, f represents fIN, b represents eIN and c represents the adjacent cortical area. C_αβγ_ is the connection parameter, where the first position α represents the afferent neurons, the second β denotes the efferent neurons, and the third position γ represents the excitatory (e) or inhibitory (i) connection. For example, C_tni_ represents the inhibitory synapse from TRN to TRC, C_tre_ denotes the excitatory synapse from retina to TRC and C_tsi_ represents the inhibitory synapse from IN to TRC. The sigmoid functions S(•) in the above Equations can transform the summated membrane potential *V*_*cell*_: *cell* ϵ{*tcr*, *in*, *trn*, *py*, *ein*, *sin*, *fin*} into the average firing rate, where 2*e*_0_ is the maximum firing rate of the neuronal population, *s_0_* is the firing threshold and *v* is the sigmoid steepness parameter. The parameters in the sigmoid function are set as *e*_0_ = 2.5s^−1^, *s*_0_ = 6mV, *v* = 0.56mV^−1^, which are presented by Jansen and Rit (1995).

Unless specially stated, the synaptic connectivity parameters and the other model parameters in Equation (1) to Equation (9) are as follows: *H*_*et*_ = 3.25mV, τ_*et*_ = 10ms, μ_*r*_ = 5sps, φ_*r*_ = 0.05sps^2^, *C*_*tre*_ = 7.1, *C*_*tpe*_ = 62, *C*_*tni*_ = 15.45, *H*_*i*_ = 22mV, τ_*i*_ = 25ms, *C*_*sre*_ = 47.4, *C*_*spe*_ = 29, *C*_*ssi*_ = 23.6, *C*_*nte*_ = 35, *C*_*npe*_ = 50, *C*_*nni*_ = 15, μ_*c*_ = 13sps, φ_*c*_ = 0.05sps^2^, *H*_*ec*_ = 2.7mV, τ_*ec*_ = 25ms, *C*_*pce*_ = 1, *C*_*pte*_ = 80, *C*_*pbe*_ = 108, *C*_*pli*_ = 33.75, *C*_*pfi*_ = 108, *C*_*bpe*_ = 135, *C*_*bte*_ = 100, *H*_*il*_ = 4.5mV, τ_*il*_ = 50ms, *C*_*lpe*_ = 33.75, *C*_*lte*_ = 40, *C*_*lfi*_ = 13.5, *H*_*if*_ = 39mV, τ_*if*_ = 10/3ms, *C*_*fpe*_ = 40.5, *C*_*fte*_ = 40, *C*_*fli*_ = 13.5. The above parameters are described by Bhattacharya et al. (2011c, 2014) and Li et al. (2020), which are on the basis of previous works (Jansen and Rit, 1995; Horn et al., 2000; Sherman and Guillery, 2001; Zavaglia et al., 2006; Jones, 2007; Sotero et al., 2007). Indicated by the work of that the recorded EEG signal within alpha band can be simulated by the sum output of the membrane potential of TRC cells (da Silva et al., 1974), the output of this model is denoted by the summated membrane potential on the TRC cell population (*V*_*trc*_).

### Simulation Method

In this work, the TCT model with DBS voltage is numerically simulated by the Euler algorithm in the Maltab2018b environment. The total simulation time is 90 s with a time step of 0.004 s. The state variables in Equation (1) to Equation (9) are initialized to zero. The epoch of the model output is drawn from the 30 s to remove the transition state. For each set of fixed parameters, the model output is averaged over 20 independent realizations to guarantee statistical accuracy. The controlling effect of DBS on AD is mainly evaluated by the relative power within theta band and alpha band. In detail, the output vector of this model is filtered between 0.5 and 50 Hz and the sampling rate is 250 Hz (Cantero et al., 2009; Bhattacharya et al., 2011c). By the FFT-based power spectral analysis, the power spectrum density is calculated using the Welch period diagram method. The relative power within each sub-band is calculated by averaging the relative power density within this band, in which the relative power density is defined as the ratio between the absolute power at each frequency and the averaged total power spectral (Moretti et al., 2004). As indicated by the reversal of EEG in a comparative test of drug effects for AD (Agnoli et al., 1983; Knott et al., 2000; Balkan et al., 2003), the degraded relative power within the theta band or the enhanced relative power within the alpha band is considered to be a notable tendency of EEG shift to normal values.

## Main Results

As stated in the Introduction, the traditional power spectral analysis especially the EEG markers have been widely used in disease differential/diagnosis, drugs safety and therapy efficacy (Jeong, 2004; Dauwels et al., 2010). The classical nonlinear dynamical methods including dynamical and bifurcation analysis have also been proposed to study neurological disorders such as epilepsy in neural mass models (Grimbert and Faugeras, 2006; Marten et al., 2009; Freyer et al., 2011). Recent works have proposed a promising “two-modal” analytical technique combining power spectral analysis with nonlinear dynamical analysis to simulate the abnormal brain oscillations in the neural mass model of the modified ARm (Bhattacharya et al., 2013, 2014) and the Hippocampal-Septal model (Zou et al., 2011, 2012). Motivated by these works, this section combines power spectral analysis and nonlinear dynamical analysis to perform the following research work. Firstly, we discuss how the neuropathological AD condition is induced by a synapse loss in this TCT model in the absence of DBS voltage. Then we systematically explore how such AD state is regulated when the DBS voltage is introduced into the neural nuclei of TRC.

### The Neuropathological Alzheimer’s Disease State Induced by Synapse Loss

**FIGURE 3:**
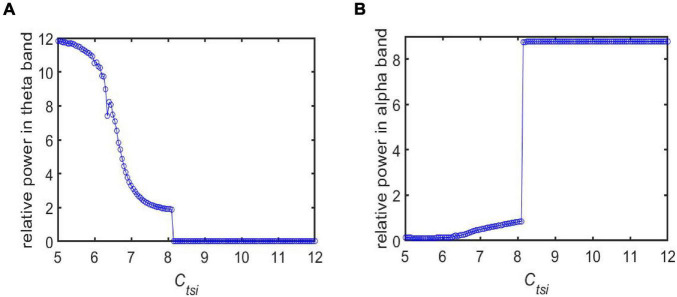
The dependence of the relative power on the synapse strength *C*_*tsi*_ for different frequency bands: **(A)** The case of relative power within the theta band; **(B)** The case of relative power within the alpha band.

Note that a loss of synapse is closely related with cognitive deficits in AD patients (Xuereb et al., 1991; Scheff et al., 2014). The loss of synapse usually leads to a decrease in the strength of synaptic connections between different neurons. Thus, in the following, we simulate how the neuropathological AD state is induced by synapse loss based on power spectral analysis and dynamical analysis. By decreasing the synaptic connectivity parameter *C*_*tsi*_ from the IN neuron population to the TRC neuron population to mimic synapse loss resulting from AD, Figures 3A,B depict the dependence of the relative power within the theta and alpha band on the connectivity parameter *C*_*tsi*_, respectively. One can observe that as *C*_*tsi*_ is decreased from 12 to 5, firstly the relative power within the theta band is fairly flat. Surprisingly it suddenly increases at a critical value of about 8.3, after which it increases steadily with a further decrease of *C*_*tsi*_. On the other hand, the relative power within the alpha band is quite steady with the initial decrease of *C*_*tsi*_. However, it decreases sharply at the critical value of about 8.3, then it decreases steadily with a further decrease of *C*_*tsi*_. The above phenomena show that an increase of the theta rhythm as well as a decrease of the alpha rhythm are consistent with the electrophysiological characteristics of EEG observed in AD patients (Coben et al., 1985; Brenner et al., 1986; Giaquinto and Nolfe, 1986; Prinz and Vitiell, 1989; Schreiter-Gasser et al., 1993; Jeong, 2004). Hence, we can conclude that the decrease of the synaptic connection strength *C*_*tsi*_ resulting from synapse loss in this TCT model can indeed simulate the neuropathological state of AD. This phenomenon accords well with the result that slowing of alpha rhythms is obtained by analyzing the peak power density within alpha band (Li et al., 2020). Note that further simulations of the relative power over the theta band and the alpha band shows decreasing other synaptic connection such as C_fpe_(PY to fIN), C_fte_(TRC to fIN) and C_lfi_ (fIN to sIN) can also induce the neuropathological AD state. The main purpose of this manuscript is to investigate how the Alzheimer’s disease can be efficiently controlled through deep brain stimulation (DBS) from the perspective of neurocomputation. For the sake of brevity, here we do not list them one by one. In the following, the neuropathological condition of AD in the TCT model is exemplified by the synapse loss in the pathway from IN to TRC.

**FIGURE 4:**
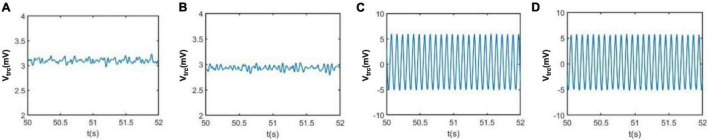
The time series plots of the model output V_*trc*_ when *C*_*tsi*_ takes some different values. From left to right, the synapse strength of *C*_*tsi*_ is successively selected as **(A)** *C*_*tsi*_ = 6.95, **(B)** *C*_*tsi*_ = 7.45, **(C)** *C*_*tsi*_ = 8.45, **(D)** *C*_*tsi*_ = 8.95.

The neural activity of the TCT model associated with the neuropathological AD state can be intuitively demonstrated using nonlinear dynamical analysis. To vividly depict the neural activity, the external inputs to the thalamic module P_1_(t) and to the cortical module P_2_(t) are simulated by Gaussian white noises. The values of mean and variance are the same as that in Figure 3, i.e., μ_r_ = 5sps, φ_r_ = 0.05sps^2^, μ_c_ = 13sps and φ_c_ = 0.05sps^2^. Figure 4 illustrates the time series plots of the model output, when the synapse strength *C*_*tsi*_takes some values of 6.95, 7.45, 8.45 and 8.95. The right two columns are for the case of *C*_*tsi*_ 8.45 and 8.95, i.e., the synapse strength is higher than the critical value of 8.3. As known that the external inputs are Gaussian white noises, the output of TCT model is noisy signal. In such case, one can observe that the output of TCT model (*denotedbyV*_*trc*_) oscillates approximately between its maximum and minimum. The left two columns are for the case of *C*_*tsi*_ 6.95 and *C*_*tsi*_ 7.45, i.e., the synapse strength is lower than 8.3. In such case, the output of TCT model oscillates slightly around a constant. As indicated in the previous work (Bhattacharya et al., 2013, 2014), the above results imply that the critical value of 8.3 looks like a bifurcation point from the viewpoint of dynamical analysis, though it cannot be completely determined by visual inspections of phase diagrams and the time series plots due to the blurring influence of external noisy inputs.

**FIGURE 5:**
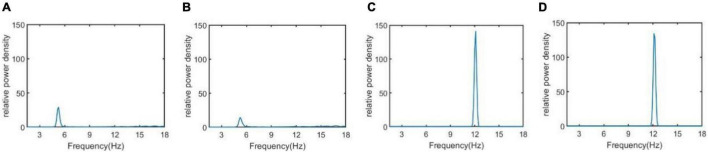
The relative power density of the thalamic module output when *C*_*tsi*_ takes some different values. From left to right, the synapse strength of *C*_*tsi*_ is successively selected as **(A)** *C*_*tsi*_ = 6.95, **(B)** *C*_*tsi*_ = 7.45, **(C)** *C*_*tsi*_ = 8.45, **(D)** *C*_*tsi*_ = 8.95.

In addition, the relative power density of the thalamic module output for *C*_*tsi*_ = 6.95, 7.45, 8.45, and 8.95 are illustrated in Figure 5, respectively. One can observe that as the connectivity *C*_*tsi*_ is decreased, there is a significant decrease of alpha rhythmic content as well as an obvious increase of theta rhythmic content, which is consistent with the relative power obtained in Figure 3. When *C*_*tsi*_ is more than the critical value of 8.3, the right two panels indicate that the model output oscillates with a dominant frequency of about 12, which has a frequency within the alpha band. When *C*_*tsi*_ is less than the critical value of 8.3, one can see that the model output visibly loses the alpha rhythmic content and has a prominent characteristic of theta rhythm.

### Regulating the Alzheimer’s Disease State by Applying Deep Brain Stimulation Voltage on Thalamic Relay Cells

Section “The Neuropathological Alzheimer’s Disease State Induced by Synapse Loss” has indicated that the neuropathological condition of AD in the TCT model can appear by a decreasing synapse strength *C*_*tsi*_. In particularly, the characteristic of AD is significant when *C*_*tsi*_ < 8.3. In this section, we introduce the DBS voltage on the neural nuclei of TRC to discuss the controlling effect of AD, when the TCT model is under such AD condition.

#### Case 1: Three parameters of DBS current are fixed

**FIGURE 6:**
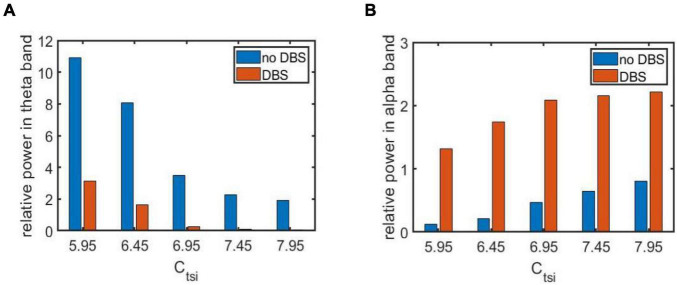
The relative power of two frequency bands before and after adding DBS current for different synapse strength under the AD pathological circumstance: **(A)** The relative power within the theta band; **(B)** The relative power within the alpha band. Here the parameters of DBS current are specific as A = 1.8*m*A,P = 0.1*s* and D = 0.008*s*.

As indicated in the above section, several synapse strength below the critical value such as *C*_*tsi*_ = 5.95, 6.45, 6.96, 7.45, and 7.95 is selected to mimic the AD pathological circumstance. Now a specific DBS current with fixed parameters A = 1.8*m*A, P = 0.1s and D = 0.008s is applied on TRC. The controlling effect of DBS is discussed by means of power spectrum analysis and nonlinear dynamical analysis. Figures 6A,B exemplify the relative power of the theta and alpha band, respectively in the case of *C*_*tsi*_ = 5.95, 6.45, 6.96, 7.45, and 7.95 when the TCT model is under the DBS voltage control. Note that the original relative power without DBS control already illustrated in Figure 3 is also included in this figure for comparison. We observe that once the DBS current is implanted into the neural nuclei of TRC the relative power within the theta band decreases prominently. On the same time, the relative power within the alpha band increases remarkably. In order to quantitatively describe these changes of relative power, the increments of relative power before and after adding DBS voltage are listed in Table 1. As shown in Table 1, the relative power of the theta band is reduced by 7.75, 7.86, 3.25, 2.14, and 1.86 after adding DBS current to TRC. Meanwhile, the relative power of the alpha band is increased by 1.19, 1.52, 1.60, 1.51, and 1.41 when the synapse connection strength *C*_*tsi*_ is 5.95, 6.45, 6.95, 7.45, and 7.95, respectively. Obviously, the reduced slow wave power within the theta band as well as the augmenting fast wave power within the alpha band is a notable tendency when EEG shift to normal values, which accords with the results of electrophysiological experiments of the reversal of the EEG of AD in a comparative test of drug effects (Agnoli et al., 1983; Knott et al., 2000; Balkan et al., 2003). This result implies that the symptom of AD is significantly relieved by applying this specific DBS current to TRC in the TCT model.

**TABLE 1:** The increments of the relative power before and after adding DBS voltage. Here the signal “+” implies that the increment is positive, otherwise the signal “–” implies that the increment is negative.

**_*Frequency band*_ *C*_*tsi*_**	**5.95**	**6.45**	**6.95**	**7.45**	**7.95**
Theta band	7.75 (−)	7.86 (−)	3.25 (−)	2.14 (−)	1.86 (−)
Alpha band	1.19 (+)	1.52 (+)	1.60 (+)	1.51 (+)	1.41 (+)

**FIGURE 7:**
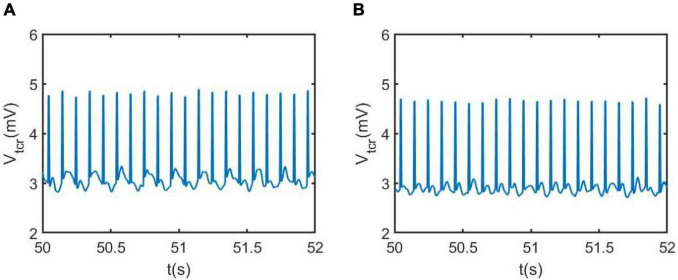
The phase diagram and the corresponding time series of the model output *V*_*tcr*_ for the thalamic module after adding DBS control when *C_tsi_* takes different values: **(A)** *C*_*tsi*_ = 6.95, **(B)** *C*_*tsi*_ = 7.45. Here the DBS parameters are A = 1.8*m*A,P = 0.1*s*, D = 0.008*s*.

**FIGURE 8:**
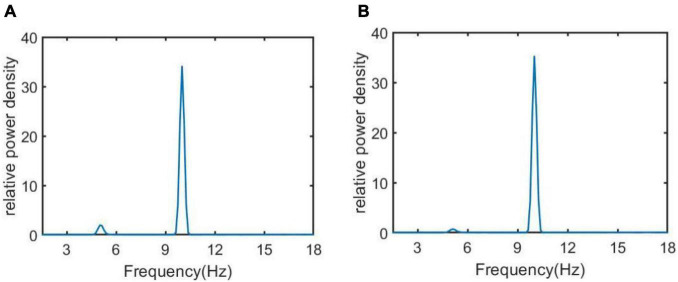
The relative power density of the thalamic module output when *C*_*tsi*_ takes some different values. From left to right, the synapse strength of *C*_*tsi*_ is successively selected as **(A)** *C*_*tsi*_ = 6.95, **(B)** *C*_*tsi*_ = 7.45.

The regulating effect of DBS on AD is further demonstrated by nonlinear dynamical analysis. Figure 7 displays the time series plots of the model output in the case of *C*_*tsi*_ = 6.95 and 7.45, when the DBS voltage is introduced into the TCT model. Compared with the result of Figure 4 (the left two panels) in which the TCT model is without DBS control, the thalamic module under DBS control restores to oscillate with large amplitude again, which are quite similar with the firing pattern showed in Figure 4 (the right two panels) that the TCT model is under normal circumstance. In addition, the relative power density of the model for *C*_*tsi*_ = 6.95 and 7.45 are, respectively illustrated in Figure 8, which indicates that the model output for the thalamic module oscillates with a dominant frequency of about 10, which has a frequency within the alpha band. Obviously, the introduction of DBS voltage into the neural nucleus of TRC indicates a notable increase of alpha rhythmic content together with a remarkable decrease of theta rhythmic content. Once more, this result confirms again that the symptom of AD is remarkably relieved by implanting the specific DBS current to TRC in the TCT model.

#### Case 2: Two parameters of DBS current are fixed

By the above analysis of relative power and dynamical system, one can conclude that the symptom of AD is significantly relieved by introducing a DBS current with the specific parameters A = 1.8*m*A, P = 0.1*s* and D = 0.008*s*. In fact, how to choose the stimulation parameters appropriately is a tricky question in animal experiments and clinical medicine. Thus, we further explore the control effect of DBS by, respectively varying the parameters of amplitude, period and duration so as to study the control effect theoretically. In the following, the synapse strength is selected as *C*_*tsi*_ = 7.45 to simulate the neuropathological condition of AD.

**FIGURE 9:**
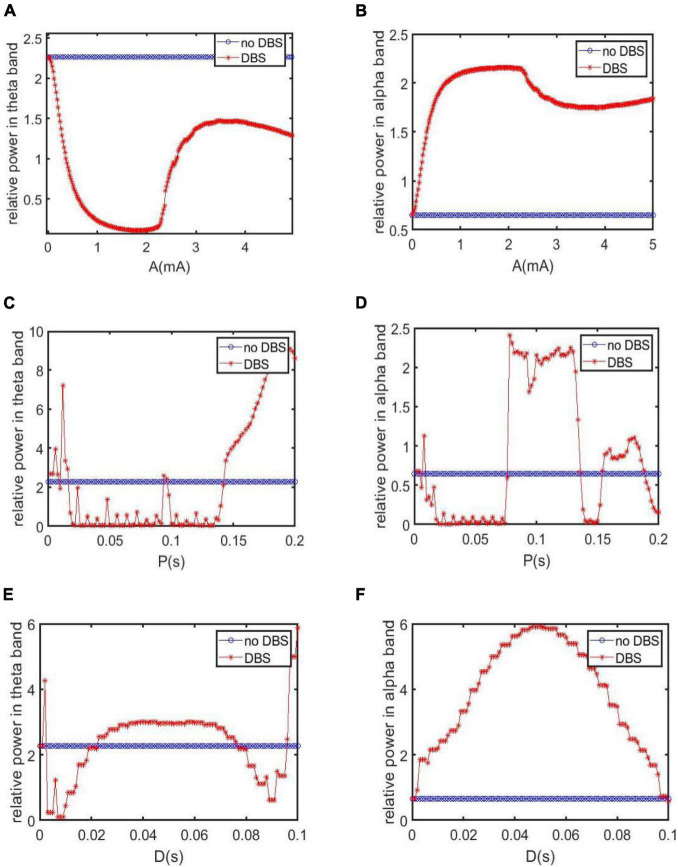
The relative power for varying stimulation parameters in different bands. For comparation, the case without DBS control is also included. **(A,B)** The dependence of the relative power within the theta and alpha band on the amplitude A when P = 0.1*s* and D = 0.008*s*. **(C,D)** The dependence of the relative power within theta and alpha band on the period P when A = 1.8*m*A, D = 0.008*s*. **(E,F)** The dependence of the relative power within theta and alpha band on the duration D when A = 1.8*m*A, P = 0.1*s*.

Firstly, when setting the DBS parameters of P = 0.1*s* and D = 0.008*s*, the control effect of DBS is investigated by varying the amplitude A. Figures 9A,B depict the dependence of the relative power on the amplitude A, in which the relative power without DBS voltage is also included for comparison. Figure 9A is for the case of the relative power within the theta band and Figure 9B is for the case of the relative power within the alpha band. On the whole, Figure 9A shows that the relative power of the theta band under DBS control is not larger than that without DBS control. In detail, when the DBS current is applied to TRC, the relative power of the theta band firstly decreases rapidly upon increasing A, reaching relative smaller value when A is in the interval of [1, 2.2]*m*A, after that it increases and decreases again with a further increase of A. On the other hand, Figure 9B displays that the relative power of the alpha band under DBS control is not smaller than that without DBS control in general. In detail, when the DBS current is applied to TRC, the relative power of the alpha band firstly increases rapidly upon increasing A, reaching a relative larger value when A is in the interval of [1, 2.2]*m*A, after that it decreases and increases again with the further increase of A. The reduced relative power within the theta band together with the augmented relative power within the alpha band, i.e., a hallmark of the reversal of the brain rhythm of AD, indicates that the symptoms of AD can be controlled by DBS current. In particularly, the control effect can be optimized by tuning the amplitude A into the interval of [1, 2.2]*m* A.

Secondly, the control effect of DBS is examined by altering the period P when fixing the DBS parameters of A = 1.8*m*A and D = 0.008*s*. The dependence of the relative power on the period P is depicted in Figures 9C,D, in which the relative power without DBS voltage is also included for comparison. Figure 9C is for the case of the relative power within the theta band. We see that when P is in the intervals of [0.0165, 0.093]s and [0.097, 0.142]s, the relative power of the theta band under DBS control has been reduced compared with the case without DBS control. Figure 9D is for the case of the relative power within the alpha band. It is clear that when P is in the intervals of [0.077, 0.136]s and [0.154, 0.188]s, the relative power of the alpha band after applying DBS current is higher than that without DBS current. From the perspective of the reversal of the brain rhythm of AD reported in the previous works (Agnoli et al., 1983; Knott et al., 2000; Balkan et al., 2003), the combined results of diminished relative theta band power and increased relative alpha band power imply that the AD symptom can be effectively controlled by tuning the period P into the interval of [0.077,0.093]s and [0.097,0.136]s.

Thirdly, we discuss the control effect of DBS on AD by altering the duration D. Here the other two parameters are chosen as A = 1.8*m*A and P = 0.1*s*. The relative power of the thalamus module in the theta band after adding DBS voltage is illustrated in Figure 9E. It can be seen that when D is in [0.003, 0.022]s and [0.077,0.095]s. the theta band relative power after adding DBS voltage is less than that without DBS control. On the other hand, as shown in Figure 9F, the relative power of the alpha band with DBS control is not smaller than that without DBS control on the whole. In view of the previous comment of the reversal of the brain rhythm of AD (Agnoli et al., 1983; Knott et al., 2000; Balkan et al., 2003), i.e., the tendency of EEG spectral analysis shifted into more normal patterns after drug treatment, the reduced relative power within theta band together with the increased relative power within the alpha band after adding DBS control indicates that the AD symptom can be effectively relieved by adjusting the duration D into the interval of [0.003,0.022]s and [0.077,0.095]s.

#### Case 3: One parameters of DBS current is fixed

In the above subsection, we have detected the effective stimulation parameter range to control AD when fixing the other two parameters of DBS current. In the following, we try to capture the effective parameter space by freezing one simulation parameter based on the analysis of the theta or alpha band relative power. Here, the synapse connection from the IN neuron population to TRC is selected as *C*_*tsi*_ = 7.45 so as to mimic a neuropathological condition of AD. As shown in Figure 3, in such case the relative powers in the theta and alpha band are 2.2641 and 0.6467, respectively.

**FIGURE 10:**
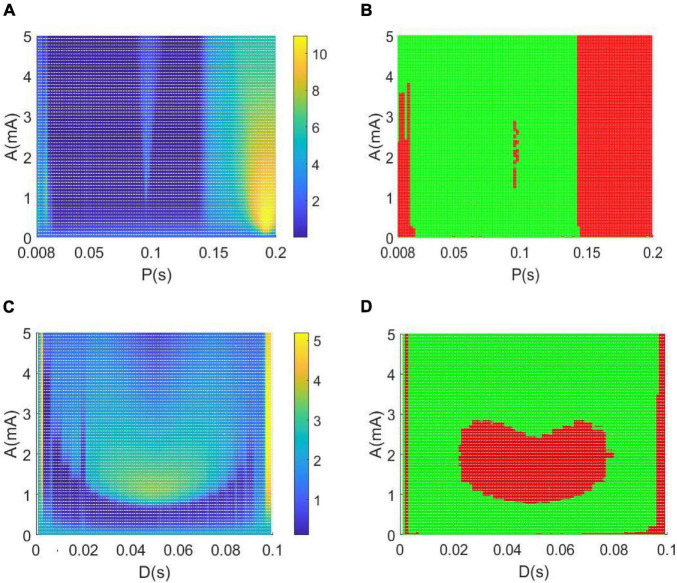
**(A,B)** The dependence of the theta band relative power on the amplitude A and period P when setting the duration *D* = 0.008 s. **(C,D)** The dependence of the relative theta band power on the amplitude A and duration D when setting the period *P* = 0.1 s. Note that the green dots in panels denote that the relative power within theta band is less than the critical value of 2.2641, meanwhile, the red dots represent that the relative power within theta band is larger than the critical value of 2.2641.

Figure 10A depicts the variations of the relative power within the theta band in the parameter space of amplitude A and period P, in which the remaining parameter of duration is set as D = 0.008*s*. To depict the control effect of DBS voltage intuitively, in Figure 10B the relative theta band power larger than the critical value of 2.2641 is marked by red dots, otherwise, the relative theta band power less than the critical value is marked by green dots. From the perspective of the reversal of the theta rhythm of AD reported in the previous works (Agnoli et al., 1983; Knott et al., 2000; Balkan et al., 2003), the diminished relative power within theta band implies that the AD symptom can be effectively controlled, when the amplitude A and period P in the parameter space are appropriately adjusted to the green region.

Similarly, when fixing the period P = 0.1*s*, the dependence of the theta band relative power on the amplitude A and duration D is illustrated in parameter space of (A, D) in Figures 10C,D. In particularly, the green dots in Figure 10D denote that the relative power within the theta band is less than the critical value of 2.2641. Meanwhile, the red dots represent that the relative power within the theta band is larger than the critical value of 2.2641. In view of the reversal of the theta rhythm of AD obtained in these works (Agnoli et al., 1983; Knott et al., 2000; Balkan et al., 2003), the decreased relative power within the theta band indicates that the AD symptom can be significantly relieved when the amplitude A and duration D are appropriately tuned to the green region.

**FIGURE 11:**
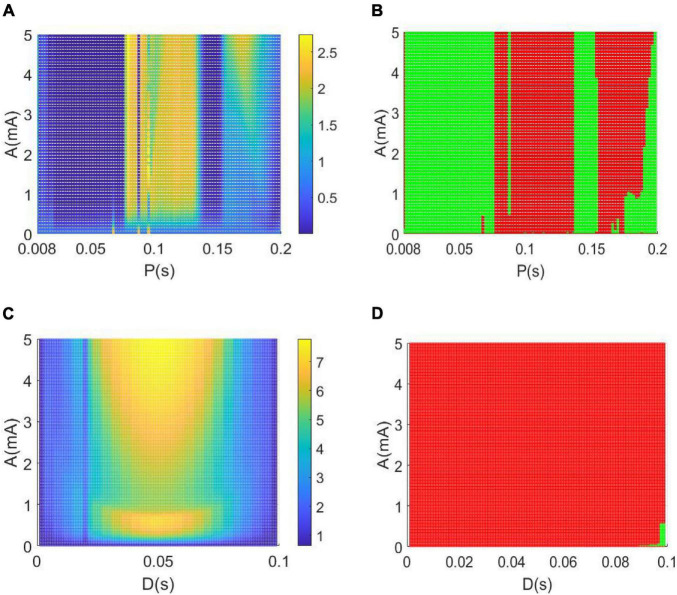
**(A,B)** The variation of the alpha band relative power with the amplitude A and period P when setting the duration *D* = 0.008 s. **(C,D)** The dependence of the relative alpha band power on the amplitude A and duration D when setting the period *P* = 0.1 s. Note that the red region denotes that the relative alpha band power is greater than the critical value of 0.6467, while the green region denotes that the relative alpha band power is less than the critical value of 0.6467.

Meanwhile, Figure 11A depicts the change of relative power within the alpha band in the parameter space of the amplitude band A and the period P when the rest parameter of the duration is set to *D* = 0.008 s. In order to intuitively describe the control effect of the DBS voltage in Figure 11B, the relative alpha band power greater than the critical value of 0.6467 is highlighted by red dots, otherwise, the relative alpha band power less than the critical value is highlighted by green dots. From the perspective of alpha rhythm reversal reported in previous studies of AD (Agnoli et al., 1983; Knott et al., 2000; Balkan et al., 2003), the increased relative power within the alpha band means that the AD symptoms can be effectively relieved when the amplitude A and the period P are appropriately adjusted to the red area in the parameter space. Similarly, the variation of the relative power within the alpha band is illustrated in the parameter space of (A, D) in Figures 11C,D when the fixed period *P* = 0.1 s. The red region denotes that the relative alpha band power is greater than the critical value of 0.6467. From the perspective of the alpha rhythm reversal of AD obtained in previous works (Agnoli et al., 1983; Knott et al., 2000; Balkan et al., 2003), the augmented relative power within the alpha band means that the AD symptoms can be significantly controlled when the amplitude A and the period P are appropriately adjusted to the red zone in the parameter space.

## Conclusion and Discussion

Based on a modified TCT model containing the nucleus of thalamus and cortex, this work aims to investigate the control effects of DBS on AD by introducing a DBS voltage to the TRC neuron population in view of neurocomputation. The neuropathological condition of AD induced by synapse loss is firstly simulated by decreasing one synapse strength*C*_*tsi*_ from the IN neuron population to the TRC neuron population. Through the analysis of power spectrum and nonlinear dynamical analysis of the thalamic module, the phenomenon that an increase of the theta rhythm together with a decrease of the alpha rhythm is detected upon decreasing synapse strength *C*_*tsi*_. These results are accordance with the characteristics of AD neuronal rhythm reported in the literature (Brenner et al., 1986; Schreiter-Gasser et al., 1993; Jeong, 2004; Bhattacharya et al., 2011c, 2013, 2014; Li et al., 2020), which indicates that the neuropathological condition of AD in this TCT model can be imitated by decreasing the synapse strength. Under such neuropathological circumstance, a specific deep brain stimulation voltage is then implanted into the nucleus of TRC in this TCT model. Through the DBS current’s regulation, not only the relative power within the theta band decreases prominently, but also the relative power within the alpha band increases remarkably. According to the comment of the reversal of EEG in a comparative test of drug effects for AD (Agnoli et al., 1983; Knott et al., 2000; Balkan et al., 2003), the reducing of the slow wave power within the theta band as well as the augmenting of the fast wave power within the alpha band is a notable tendency of EEG shift to normal values. Meanwhile, the thalamic module restores to oscillate with large amplitude, just like the firing pattern under normal circumstances. These results demonstrate that the symptoms of AD can be significantly relieved by applying this specific DBS voltage to the neural nucleus of TRC in the TCT model. The therapeutic effects of DBS control on AD are further examined in various parameter space so as to detect the suitable range of stimulation parameters. The obtained results illustrate that the controlling effect of DBS on AD can be efficient by appropriately adjusting the key parameters of the DBS current including amplitude A, period P and duration D. The efficient parameter regions controlling AD, showed in Figures 10, 11, are various in different parameter spaces. This is helpful for flexible selection of DBS parameters to achieve optimal DBS setting for different subjects with different AD severity. Meanwhile, some efficient parameter regions contain large amplitude A and small period P, which may lead to more DBS signal power in general to achieve therapeutic effectiveness. We hope this work would provide a good theoretical basis to formulate optimal DBS setting for different subjects individually. On the same time, it may offer a potential target of thalamus for DBS in AD in future clinical trials.

At last, we point that the main purpose of this work is to explore the therapeutic effects of DBS on AD from the perspective of neurocomputation, in which we introduce the DBS voltage into the neural nucleus of TRC in the modified TCT model. As stated in section “A Modified Thalamo-Cortico-Thalamic Model With Deep Brain Stimulation Voltage,” the TCT model includes a thalamus module and a cortical module. The employment of this TCT model is to vividly depict the plausible thalamo-cortico-thalamic loop with more important biologically synaptic ingredients. One may wonder the controlling effect of DBS on AD when the TCT model is simplified. For example, the considered model is the modified ARm (which only includes TRN and TRC), and the DBS voltage is into the neural nucleus of TRC. Our further simulation indicates that the DBS with appropriate parameters of A, P and D can also efficiently relieve the AD symptom resulting from the decrease of synapse connectivity from retina to TRC or TRN to TRC.

In addition, as stated in section “The Neuropathological Alzheimer’s Disease State Induced by Synapse Loss,” loss of synapse is closely related with cognitive deficits in AD patients. The loss of synapse usually leads to a decrease in the strength of synaptic connections between different neurons. At present, this work focuses on the neuropathological AD state induced by decreasing the synaptic connectivity parameter, so as to mimic synapse loss resulting from AD. In fact, the EEG marker of AD patients, e.g., a slowing of alpha rhythm, may be greatly influenced by synapse connectivity parameters. In different neurocomputation models, the phenomenon of slowing of alpha rhythm can be induced not only by increasing synapse connectivity but also by decreasing synapse connectivity, which is dependent on parameter range when considering a relatively large parameter interval. In some subinterval, decreasing synapse connectivity to a certain range may induce a slowing of alpha rhythmic content. In other subinterval, increasing synapse connectivity up to a certain range may also induce a slowing of alpha rhythmic content. This is a very interesting question deserved to be deeply investigated in the future.

## Data Availability Statement

The original contributions presented in the study are included in the article, further inquiries can be directed to the corresponding author/s.

## Author Contributions

XY, RZ, ZS, and JK conceived the idea of the study, analyzed the results, and drafted and revised the manuscript. XY and RZ planned and performed numerical simulations. All authors discussed the results and revised the manuscript.

## Conflict of Interest

The authors declare that the research was conducted in the absence of any commercial or financial relationships that could be construed as a potential conflict of interest.

## Publisher’s Note

All claims expressed in this article are solely those of the authors and do not necessarily represent those of their affiliated organizations, or those of the publisher, the editors and the reviewers. Any product that may be evaluated in this article, or claim that may be made by its manufacturer, is not guaranteed or endorsed by the publisher.
